# The impact of a best practice advisory on inpatient prescribing of piperacillin-tazobactam

**DOI:** 10.1017/ash.2022.12

**Published:** 2022-03-07

**Authors:** Katherine L. Peterson, Bradley J. Tompkins, John W. Ahern, Lindsay M. Smith

**Affiliations:** 1 Division of Infectious Disease, University of Vermont Medical Center, Burlington, Vermont; 2 Department of Medicine, Larner College of Medicine at The University of Vermont, Burlington, Vermont; 3 Department of Pharmacy, University of Vermont Medical Center, Burlington, Vermont

Successful antibiotic stewardship programs (ASPs) have been shown to significantly improve antimicrobial prescribing in acute-care hospitals.^1,2^ Historically, data regarding which interventions are most effective have been limited.^3,4^

The University of Vermont Medical Center (UVMMC) is an academic medical center located in Burlington, Vermont. The ASP at UVMMC publishes institutional guidelines regarding empiric antibiotic selection based on the local antibiogram and national guidelines. In a medication-use evaluation of piperacillin-tazobactam, ∼66% of piperacillin-tazobactam prescriptions did not follow the UVMMC guidelines. This finding prompted the ASP to build a best practice advisory (BPA) in the electronic medical record (EMR) to function as an antibiotic timeout (ATO). We evaluated the impact of the BPA on the rate of piperacillin-tazobactam consumption as measured by defined daily dose (DDD) per 1,000 patient days.

## Methods

The BPA activated when the orders tab was opened in the EMR once a patient had received piperacillin-tazobactam for 72 hours. Clinicians had to select an option to continue working in the patient’s chart: (1) cultures reviewed, continue piperacillin-tazobactam, (2) defer to primary team, (3) cultures will be reviewed at a later time, or (4) cultures reviewed, discontinue piperacillin-tazobactam. The BPA would appear again if options 2 or 3 were selected. No additional interventions to encourage alterations in the current antimicrobial therapy were underway during the study period.

Antibiotic administration data were extracted on all hospitalized, nonpregnant adults who received piperacillin-tazobactam, cefazolin, ceftriaxone, or meropenem between July 1, 2017, and June 19, 2018 (the pre-BPA period) and between July 1, 2018, and June 19, 2019 (the BPA period). Patients who received preoperative antibiotics were excluded. The University of Vermont Institutional Review Board approved this study.

The data were summarized on the visit level and were converted to monthly DDD per 1,000 patient days. An interrupted time series analysis was performed using Stata version 16.0 software (StataCorp, College Station, TX) to evaluate for changes in antibiotic consumption over the study periods. Statistical significance was defined as *P* < .05.

**Fig. 1.**
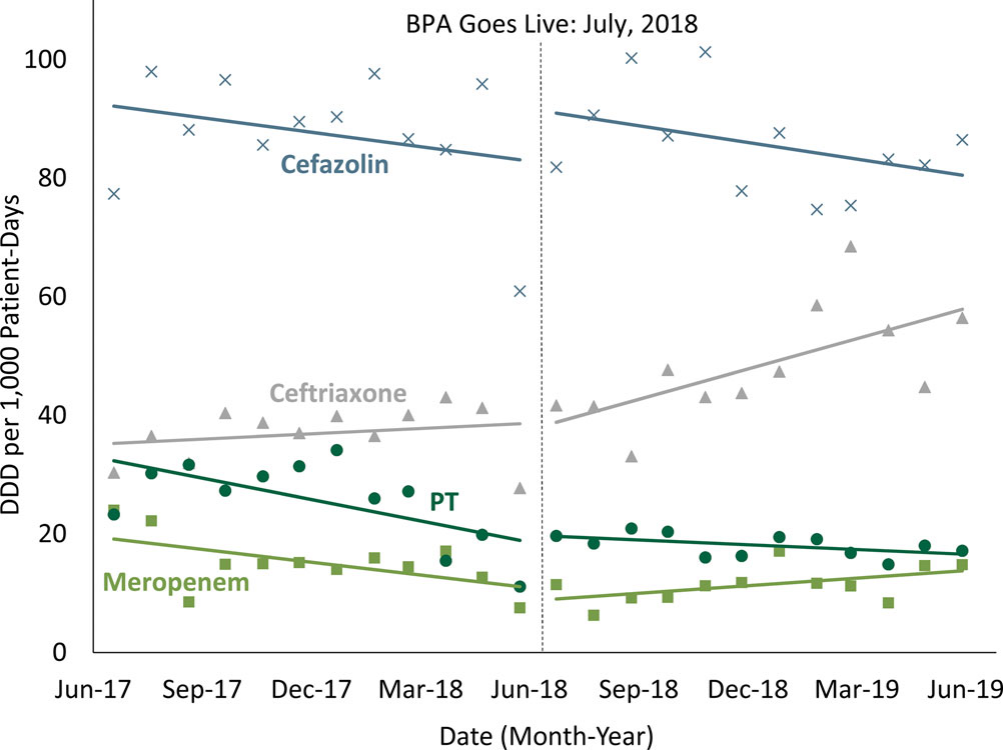
Interrupted time series showing defined daily dose (DDD) per 1,000 patient days of cefazolin, ceftriaxone, piperacillin-tazobactam, and meropenem before and after implementation of the best practice advisory (BPA).

## Results

In total, 7,094 patients (8,077 visits) were included in the pre-BPA group, and 6,661 patients (8,399 visits) were included in the BPA group. Piperacillin-tazobactam was administered to 795 patients (1,019 visits) and 528 patients (733 visits) in the pre-BPA and BPA periods, respectively. The BPA appeared 1,478 times across 510 visits.

In the pre-BPA period, piperacillin-tazobactam usage declined at a rate of −1.22 DDD per month (*P* = .04). Over the same period, cefazolin, ceftriaxone, and meropenem administration did not significantly change.

In the BPA period, piperacillin-tazobactam usage continued to decline at a rate of −0.27 DDD per month (*P* = .02). Cefazolin usage did not significantly change, but both ceftriaxone (1.73 DDD) and meropenem (0.43 DDD) usage increased significantly.

When comparing the pre-BPA and BPA periods, the usage trends for piperacillin-tazobactam, cefazolin, and ceftriaxone did not differ significantly. The change in usage trends for meropenem was found to be statistically significant (*P* = .02).

## Discussion

We detected no significant difference in the rate of decline of piperacillin-tazobactam utilization between the pre-BPA and BPA periods. The data do not support the use of a BPA to serve as an ATO to facilitate improvement in prescribing of piperacillin-tazobactam at our institution.

Data supporting ATO designs or clinical scenarios in which ATOs may be most effective are limited.^5^ Literature review yielded few studies that specifically examined the use of BPAs as a method of completing an ATO. Most studies used provider-driven ATOs or had incorporated provider audit and feedback (PAF). Notably, our BPA was not accompanied by PAF.

This study had several limitations. Our BPA may not have affected piperacillin-tazobactam utilization due to several confounding factors. First, our institutional piperacillin-tazobactam usage was already decreasing in the pre-BPA period, which likely made it difficult to detect a difference during the BPA period. Second, the results of the medication-use evaluation were widely distributed via e-mail in March of 2018 and encouraged clinicians to use ceftriaxone with metronidazole over PT in the absence of an indication for antipseudomonal therapy. Third, the results of the meta-analysis by Hammond et al^6^ may have influenced clinicians to shift empiric antipseudomonal therapy away from PT, independent of our intervention, due to concern for acute kidney injury when used in conjunction with vancomycin.^6^ We observed an increase in meropenem usage, and we speculate that this finding may be related. Additional antipseudomonal drug administration data were not available for analysis. Fourth, the stewardship features of our EMR were limited at the time of the study. As a result, the BPA could not be programmed to target only the primary team, to link culture data, or to notify the ASP of a BPA trigger for PAF follow-up. Lastly, our study was limited by its retrospective design and only included a single academic medical center.

Nonetheless, our experience is valuable in that it shows the limitation of using a BPA to function as an ATO, independent of other stewardship strategies, in the context of complex decision making and busy practice environments. Despite the limitations of our study, our findings support the Centers for Disease Control and Prevention’s latest recommendations that ASPs should prioritize PAF and preauthorization over ATOs.^1^ We recognize the value of regular assessments of antimicrobial necessity and appropriateness by clinical teams, but our data do not support the use of a BPA functioning as an ATO as an effective way to complete this task. Therefore, we have decided to remove the BPA from our EMR.
